# FLASH Knockdown Sensitizes Cells To Fas-Mediated Apoptosis via Down-Regulation of the Anti-Apoptotic Proteins, MCL-1 and Cflip Short

**DOI:** 10.1371/journal.pone.0032971

**Published:** 2012-03-09

**Authors:** Song Chen, Hedeel Guy Evans, David R. Evans

**Affiliations:** 1 Department of Biochemistry and Molecular Biology, Wayne State University School of Medicine, Detroit, Michigan, United States of America; 2 Department of Chemistry, Eastern Michigan University, Ypsilanti, Michigan, United States of America; National Center for Scientific Research Demokritos, Greece

## Abstract

FLASH (FLICE-associated huge protein or CASP8AP2) is a large multifunctional protein that is involved in many cellular processes associated with cell death and survival. It has been reported to promote apoptosis, but we show here that depletion of FLASH in HT1080 cells by siRNA interference can also accelerate the process. As shown previously, depletion of FLASH halts growth by down-regulating histone biosynthesis and arrests the cell cycle in S-phase. FLASH knockdown followed by stimulating the cells with Fas ligand or anti-Fas antibodies was found to be associated with a more rapid cleavage of PARP, accelerated activation of caspase-8 and the executioner caspase-3 and rapid progression to cellular disintegration. As is the case for most anti-apoptotic proteins, FLASH was degraded soon after the onset of apoptosis. Depletion of FLASH also resulted in the reduced intracellular levels of the anti-apoptotic proteins, MCL-1 and the short isoform of cFLIP. FLASH knockdown in HT1080 mutant cells defective in p53 did not significantly accelerate Fas mediated apoptosis indicating that the effect was dependent on functional p53. Collectively, these results suggest that under some circumstances, FLASH suppresses apoptosis.

## Introduction

**Figure 1:**
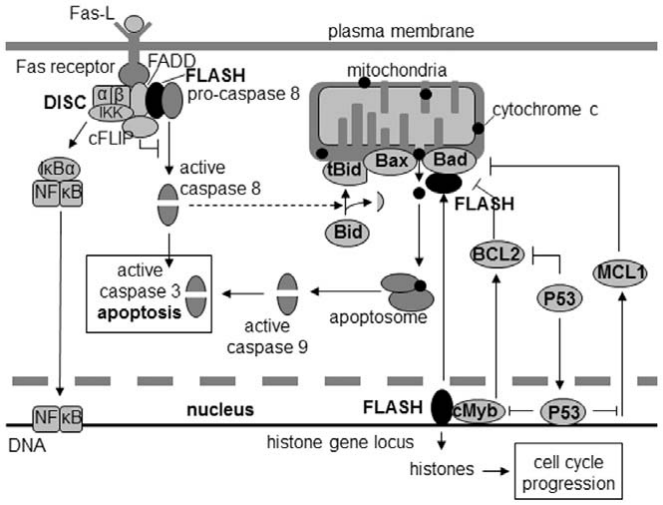
The role of FLASH in the apoptotic pathways. In the extrinsic pathway, the Fas ligand (FasL) binds to the Fas receptor and triggers the assembly of the DISC complex. FLASH binds pro-caspase 8 and translocates to the DISC complex where it associates with FADD. Active caspase-8 is formed at the DISC by proteolytic cleavage. The active caspase then cleaves and activates the executioner protease, caspase-3. c-FLIP short is also part of the DISC and inhibits the activation of caspase-8. Caspase-3 is also activated in the intrinsic or mitochondrial pathway triggered by a variety of apoptotic signals that culminate in the formation of pores that allow the release of cytochrome c. Cytochrome c associates with Apaf-1 forming the apoptosome which recruits and activates pro-caspase 9, which in turn activates pro-caspase 3. The translocation of FLASH from the nucleus to the mitochondria is thought to be one of the signals that initiate the mitochondrial apoptotic pathway. The extrinsic and intrinsic pathways are linked by Bid, a cytoplasmic proapoptotic protein that is cleaved by caspase-8 generated at the DISC complex. Once cleaved, the truncated Bid (tBid) migrates to the mitochondria where it interacts with Bax and Bad, proteins that promote mitochondrial permeability and cyctochrome c release. FLASH also binds to the histone gene locus where it participates in processing the histone mRNA that is necessary for S-phase progression. FLASH is also a coactivator of c-Myb which controls the expression of several proteins that play a role in proliferation, including the anti-apoptotic protein, BCL-2. P53 down regulates the expression of BCL-2 and another pro-apoptotic protein, MCL-1.

FLASH (CASP8AP2) is a large multifunctional protein that has been implicated in many different cellular processes including apoptosis, histone mRNA processing, S-hase progression, NF-kappa B activation and the regulation of transcription. In 1999, Imai et al. [1] discovered a 220 kDa protein, which they designated FLICE associated huge protein or FLASH, since it associates with caspase-8 and promotes Fas induced apoptosis. There are two major apoptotic pathways. The binding of ligands to the FAS receptor, a member of the TNF family of plasma membrane receptors, triggers the assembly of the death inducing signaling complex (DISC) (Figure 1). Imai et al. [1] showed that in 293 T cells, FLASH associates with the adaptor protein, FADD, recruiting caspase-8 to the activated DISC. Oligomerization of FLASH results in the proteolytic cleavage and activation of caspase-8. Caspase-8 in turn activates other caspases including the executioner protease, caspase-3.

In the intrinsic or mitochondrial apoptotic pathway several intra- and extracellular apoptotic signals induce the release of proteins from the mitochondria including cytochrome c (Figure 1). Cytochrome c associates with the apoptotic protease activating factor 1 (APAF-1) to form the apoptosome. The recruitment of pro-caspase-9 molecules to the apoptosome promotes its proteolytic activation which leads to the activation of the downstream executioner, caspase-3. The mitochondrial apoptotic pathway also serves to amplify the apoptotic response triggered by the activation of the Fas receptor [2].

The response to stimulation of the Fas receptor differs according to cell type [3]. Type I cells such as SKW6.4 and H9 cells quickly assemble large amounts of DISC upon binding of the Fas ligand with the rapid activation of caspase 8 and caspase 3. Very little DISC is formed upon stimulation of Type II cells such as CEM and Jurkat cells. However, sufficient caspase-8 is activated to cleave the cytoplasmic protein, Bid. Truncated Bid, tBid, relocalizes to the mitochondria where it binds to Bak/Bax which together with Bad promote the formation of mitochondrial pores and the release of cytochrome C. The loss of the mitochondrial membrane potential occurs prior to the activation of caspase-3 and caspase-8. Thus, the mitochondrial pathway is indispensible for type II cells to undergo apoptosis.

FLASH was originally thought [1] to be exclusively a cytoplasmic protein but more recent studies showed that it is primarily nuclear and that it is localized within a variety of discrete nuclear bodies. FLASH was identified [4] as an indispensible component of Cajal bodies, small nuclear organelles involved in numerous cell functions. RNA interference showed that depletion of FLASH resulted in disruption of Cajal body structure and relocation of its components. In other studies, FLASH was found [5], [6] to be primarily localized in promyelocytic leukemia nuclear bodies which are involved in apoptosis, the regulation of senescence and tumor suppression. FLASH associates with Sp100 [5], [7], an essential PML component. Although PMLs are distinct nuclear bodies, they are often found in association with Cajal bodies and other nuclear organelles. Immunofluorescence microscopy [8] showed that FLASH was 100% coincident with NPAT, the nuclear protein localized near histone locus bodies [9] on chromosome 6 and 12. HLBs are often associated with but are not identical to the coilin containing Cajal bodies, although the two organelles co-localize during the S phase of the cell cycle. These authors [8], [10] did not find FLASH in other nuclear bodies such as nuclear speckles or PML bodies.

In 2007, Milovic-Holm et al. [7] made the intriguing observation that activation of the Fas receptor triggers the translocation of FLASH from the PML nuclear bodies to the cytoplasm, where it associates with caspase-8 at the mitochondrial surface, thereby activating the mitochondrial apoptotic pathway. Leptomycin B, an inhibitor of Crm1-dependent nuclear export, blocked egress of FLASH from the nucleus and prevented mitochondrial damage. Caspase-8 was nevertheless still activated, albeit to a lesser extent, presumably at the DISC assembly. FLASH depletion by siRNA interference followed by induction of the Fas receptor with anti-Fas antibodies for 7 hours resulted in a 57% decrease in apoptosis. A recent study [11] may provide insight into the translocation mechanism. FLASH was shown to form a complex with Ro52, an E3 ubiquitin ligase that moves along cytoplasmic microtubular networks. Simultaneous overexpression of Ro52 and FLASH induces the relocation of another apoptotic protein, DAXX, from the nucleus to the cytoplasm.

Barcaroli et al. [8], [10] discovered that depletion of FLASH by RNA interference abolished histone biosynthesis and induced cell cycle arrest in S phase. It was subsequently shown [12] that FLASH is necessary for proper processing of the 3′-end of the histone pre-mRNA. FLASH also plays a significant role in the transcriptional regulation of histone genes. The FLASH binding partner, NPAT (p220), is an activator of histone gene transcription [9], [13] under the control of cyclin E/Cdk2 kinase. Moreover, CHIP assays demonstrated that FLASH interacts with histone gene promoter sequences [8]. The interaction of FLASH with the arsenite resistance protein, Ars2, a protein involved in the formation of microRNA, was shown [14] to be indispensible for cell cycle progression. Similar results [15] were observed during embryogenesis where FLASH cooperates with the transcription factor p73 to regulate histone gene transcription and cell cycle progression. These authors also found that FLASH knockout is lethal in embryonic mice.

FLASH is also a co-activator of c-Myb, a transcription factor normally associated with growth and survival. Both proteins colocalize at active transcription loci [16], [17]. The enhancement of transcriptional activity by FLASH is comparable with that obtained with the c-Myb co-activator, P300. The E3 SUMO-protein ligase, PIAS1, was also found to interact with FLASH and enhance its transcriptional activity and the expression of genes under control of c-Myb [17]. In some instances, FLASH was found to repress transcription. It binds to and inhibits the activity of the p160 nuclear receptor coactivator (GRIP 1) thus suppressing the expression of the glucocorticoid receptor [18] in human colon carcinoma cells. In contrast, it enhances transactivation of both the glucocorticoid and mineralocorticoid receptors in mouse hippocampal cells but had only a small repressive effect in neuroblastoma cells [19]. FLASH also modulates the activity of the transcription factor NF-kappa B via a TRAF-2 dependent pathway [20], [21] Depletion of FLASH by RNA interference abolishes the activation of NF-kappa B, while overexpression of FLASH activates its activity in a dose dependent manner.

Thus, FLASH is involved in several pathways related to cell death, growth and survival. Those studies [1], [7] that specifically examined its role in cell death, suggest that it promotes apoptosis. We report here that under certain circumstances, it can also effectively suppress apoptosis.

## Materials and Methods

### Antibodies and Reagents

Antibodies used for this study were rabbit anti-FLASH (Bethyl Laboratories, Montgomery, TX), mouse anti-caspase-8 (9746), rabbit anti-caspase-3 (9662), rabbit anti-cleaved caspase-3 (9664), rabbit anti-poly (ADP-ribose) polymerase, PARP (9542), rabbit anti-cleaved PARP (5625), rabbit anti-MCL-1 (5453) (Cell Signaling, Beverly, MA); mouse monoclonal β-tubulin (sc-5274), mouse monoclonal anti-p53 (sc-126), rabbit anti-FLASH M300 (sc-9088), mouse anti-NPAT (sc-136007) and mouse anti-PML (sc-966), mouse anti-FLIPS/L (sc-5276), rabbit anti-IKKα (sc7607) (Santa Cruz Biotechnology, Santa Cruz, CA); rabbit anti-histone H3 (21137) (Signalway Antibody, Pearland, TX), rabbit anti-HDAC1(10197-1-AP), rabbit anti-coilin (10967-1-AP) and rabbit anti-p21(10240-1-AP) (Proteintech Group, Inc). A panel of caspase inhibitors (FMKSP01) and recombinant human Fas Ligand/TNF9SF (126-FL-010) were purchased from R&D (Minneapolis, MN). MG132, cycloheximide (CHX), actinomycin D were from Sigma and adriamycin was from Santa Cruz. Staurosporine (STS) was from Invitrogen (Carlsbad, CA).

### Cell Culture and Induction of Apoptosis

HT1080 cells (wild type p53) and HT1080-6TG (mutant p53) (a gift of Dr Eric J Stanbridge, Department of Microbiology and Molecular Genetics, University of California, Irvine, CA) and HeLa cells (ATCC) were cultured in DMEM containing 10% fetal bovine serum, 100 units/ml penicillin, 100 µg/ml streptomycin, in 5% CO_2_ at 37°C. The MCF10A cell line, obtained from Drs. Santner and Pauley (Karmanos Cancer Institute, Wayne State University, Detroit, MI), was cultured according to the original publications [22], [23]. For the induction of apoptosis, the cells were incubated with 100 ng/ml of the recombinant Fas ligand (R&D, Minneapolis, MN) or 1 µg/ml of the agonist mouse monoclonal anti-human CD95 (Fas) antibody (Invitrogen, AHS9552) for the indicated periods of time in 5% CO_2_ at 37°C. Alternatively, apoptosis was induced by incubating the cells with 1 µM staurosporine. The progression through apoptosis was monitored by measuring the activation of the caspases and the cleavage of PARP. The distribution of cells in different phases of the cell cycle was measured using a Becton-Dickinson FACScan cytofluorometer at the Wayne State University, Karmanos Cancer Institute, Flow Cytometry Facility.

### SiRNA Interference

HT1080 cells were grown in 6 well plates to 20–30% confluence. Cells were transfected with siRNA directed against the FLASH mRNA and, as a negative control, with a scrambled siRNA, using RNAi Lipofectamine RNAiMAX (Invitrogen, Carlsbad, CA), according to the manufacturer's protocol. The oligonucleotides used for these studies were purchased from Invitrogen (Carlsbad, CA). 1) FLASH Stealth RNAi™ siRNA HSS115171: GAAACAGAAUGAACCAAAGACUGAU; 2) FLASH Stealth RNAi™ siRNA HSS115172: GAAAGCUGAGAGUGGUCCAAAUGAA; 3) FLASH Stealth RNAi™ siRNA HSS115173: CCUGUGGUAAUGGAUGUAUUACAAA. To assess the extent to which the expression of FLASH was suppressed, cell extracts were isolated at various times following transfection and the cell lysate was analyzed by Western blotting. Equivalent amounts of total protein were analyzed as determined by the Lowry method using bovine serum albumin (BSA) as a standard. Immunoblotting of β-tubulin or β-actin was used to verify that equal amounts of total protein had been loaded on the gel. The same procedure was used to suppress p53 expression using a commercial siRNA of proprietary sequence (Santa Cruz, sc-29435) and siRNA against coilin was purchased from Invitrogen (HSS112012).

### Western blot Analysis

Total cell extracts were prepared in a lysis buffer containing 20 mM Tris-HCl, pH 7.5, 137 mM NaCl, 1% Triton X-100, 10% glycerol, 0.2 mM PMSF supplemented with a 1× cocktail of phosphatase and protease inhibitors (Sigma). Protein samples were heated at 95°C for 10 min and separated by SDS-PAGE using 4–12% gradient gel and transferred onto a nitrocellulose membrane. Western blots were developed using the Western Lighting Plus-ECL reagent (NEL104001EA, Perkin Elmer, Waltham, MA).

### Cell Fractionation

Cytoplasmic and nuclear fractions were isolated using the Qproteome Nuclear Protein Kit (Qiagen) according to the manufacturer's protocol. The purity of the fractions was confirmed by Western Blotting using anti-PARP, anti-HDAC1 (nuclear markers) and anti-β-tubulin (cytoplasmic marker) antibodies.

### Immunofluorescence microscopy

Cells grown on cover slips in 6-well plates were fixed with cold methanol at −20°C for 10 min and then blocked with 3% BSA in PBS for 1 h at room temperature. Cells were incubated with the primary antibody, rabbit anti-FLASH M-300, mouse anti-NPAT monoclonal antibody or mouse anti-PML monoclonal antibody, alone or in combination, overnight at 4°C. The cells were then incubated with chicken anti-rabbit IgG antibody conjugated with Alexa Fluor 594 (1∶2,000) and a chicken anti-mouse IgG antibody conjugated with Alexa Fluor 488 (1∶2,000) (Molecular Probes, Invitrogen, Carlsbad, CA) as secondary antibodies for 1 h at room temperature. After extensive washing with PBS, the cells were counterstained with Hoechst 33342, mounted and visualized using a Leica TCS SP5 Laser Scanning Confocal Microscope (Karmanos Cancer Institute Imaging and Cytometry Resources Core Facility). The images were analyzed using the Leica LAS AF Imaging software.

## Results

### Intracellular localization of FLASH

**Figure 2:**
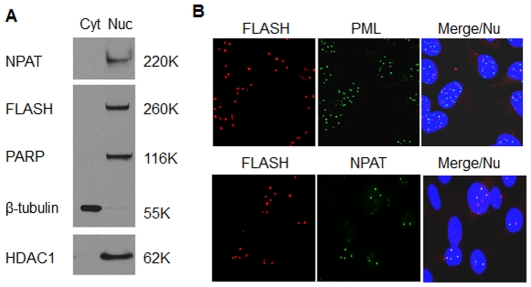
FLASH was found in the nucleus co-localized with NPAT. (**A**) HT1080 cells (5×10^6^) were fractionated into cytoplasmic (Cyt) and nuclear (Nuc) fractions (*Materials and Methods*). The fractions were analyzed by immunoblotting using antibodies directed against FLASH, NPAT, PARP, HDAC1 and β-tubulin. (**B**) immunofluorescence co-localization (*Materials and Methods*) of FLASH and PML or NPAT. HT1080 cells were fixed with cold methanol for 10 minutes, blocked, and incubated with rabbit anti-FLASH and mouse anti-PML antibodies or mouse anti-NPAT antibodies at 4°C overnight. Cells were then washed 3 times and incubated at room temperature for 1 hour with a 1/2000 dilution of the secondary antibodies, Alexa Fluor 594–conjugated anti-rabbit IgG (red) and an Alexa Fluor 488–conjugated anti-mouse IgG antibody (green). The cells were also stained with Hoechst 33342 (blue).

In agreement with previous studies [6], [7], [10], cell fractionation showed that FLASH was localized exclusively in the nuclear extract (Nuc) in the absence of apoptotic signals (Figure 2A). The purity of the cell fractions was assessed by Western blotting of the nuclear marker proteins, poly ADP-ribose polymerase (PARP), the nuclear protein ataxia-telangiectasia locus (NPAT) and histone deacetylase I (HDAC1) and the cytoplasmic marker β-tubulin. There was little or no cross contamination of nuclear and cytosolic fractions.

Within the nucleus, FLASH has variously been reported to be associated with Cajal bodies [4], PML bodies (Promyelocytic leukemia nuclear bodies) [6], [7] and in histone gene clusters [8]. In HT1080 cells, immunofluorescence microscopy showed (Figure 2B) that FLASH was concentrated in a relatively small number of discrete foci within the nucleus. In agreement with the cell fractionation results, no FLASH could be detected in the cytoplasmic compartment. When the cells were co-stained with antibodies directed against PML, there was little colocalization of FLASH and PML. In contrast, staining the cells with antibodies directed against, NPAT, a major component of the histone cluster loci, there was 100% overlap. Each of the 2–4 NPAT histone gene loci also contained FLASH, although there were additional non-overlapping FLASH foci present in other nuclear bodies.

**Figure 3:**
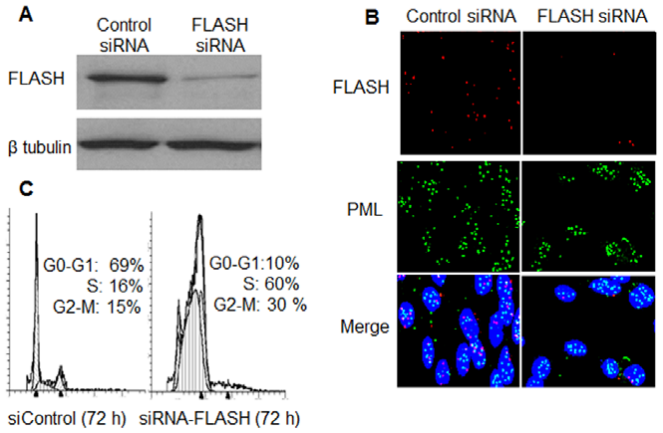
siRNA silencing of FLASH expression. (**A**) HT1080 cells were transfected with FLASH siRNA and scrambled siRNA (Control) (*Materials and Methods*). After 72 hours, the extracts of the transfected cells were analyzed by immunoblotting using FLASH antibodies and as a loading control, β-tubulin antibodies. (**B**) HT1080 cells were transfected with either a scrambled siRNA (left, Control) or a specific siRNA directed against FLASH (right). The cells were fixed with cold methanol for 10 minutes after 72 hours transfection, blocked, and incubated with rabbit anti-FLASH and mouse anti-PML at 4°C overnight. After washing three times, the cells were incubated with the secondary antibodies as described in the legend to Figure 3. The cell nucleus was stained with Hoechst 33342. (**C**) Flow cytometry analysis showed that after FLASH knockdown, cells were blocked in S phase. HT1080 cells were transfected with siRNA against FLASH or scrambled RNAi for 72 hours. The cells were trypsinized, washed with cold PBS, fixed with 70% ethanol, treated with RNase A and stained with 50 µg/ml propidium iodide. The DNA content was analyzed using a Becton-Dickinson FACScan cytofluorometer.

Three oligonucleotides complementary to different regions of the FLASH mRNA were used to silence the expression of FLASH. All three reduced the intracellular concentration of FLASH by at least 90% as determined by Western blotting of whole cell extracts (Figure 3A). Cells subjected to RNA interference with each siRNA exhibited the same phenotype with no detectable off target effects. Immunofluorescence microscopy (Figure 3B) showed that a comparable percentage of the cells lacked FLASH. In contrast, there was no effect on the number or distribution of nuclear PML bodies, as detected with anti-PML antibodies, when the expression of FLASH was suppressed.

### FLASH is necessary for cell cycle progression in HT1080 cells

As reported previously [8] in other cell lines, depletion of FLASH also caused cell cycle arrest in S phase (Figure 3C) in HT1080 cells, presumably due to the reduction of histone gene expression. In cells transfected with the scrambled siRNA control for 72 hours, 69% were found in G0–G1 and 16% in S phase. In contrast, 10% are in G0–G1 phase and 60% of the cells are in S phase in cells transfected with FLASH siRNA. Thus, the cells can progress through the G1/S check point, but cannot exit S phase. Similarly, FLASH knockdown resulted in S-phase arrest in HeLa, MCF10A and MCF-7 cells (data not shown).

### Silencing FLASH gene expression accelerates the onset of apoptosis

**Figure 4:**
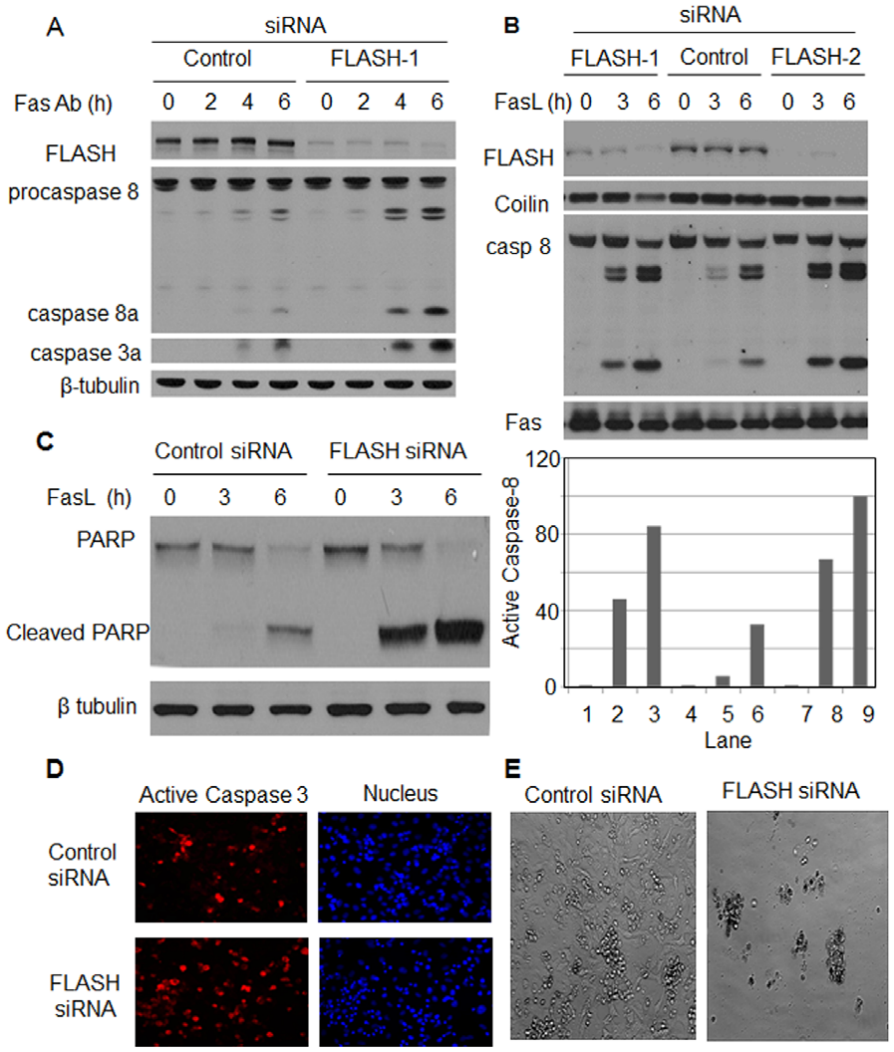
Effect of FLASH knockdown on apoptotic progression. (**A**) HT1080 cells transfected with control siRNA or with siRNA directed against FLASH were stimulated with mouse anti-Fas antibody (1 µg/ml) following the standard protocol (*Materials and Methods*) for the indicated times. The cell lysates were subjected to western blotting using anti-FLASH, anti-caspase 8, anti-cleaved caspase 3 and as a loading control, anti-β-tubulin antibodies. (**B**) HT1080 cells were transfected with two different FLASH siRNAs (FLASH-1 and FLASH-2) and the scrambled siRNA (Control) for 48 hours and then treated with 100 ng/ml FasL for the indicated times. The cell lysates were subjected to immunoblotting using FLASH, PARP, caspase 8, coilin and Fas antibodies. The developed blot was scanned to determine the relative levels of active caspase-8 shown in the bar graph. (**C**) A time course showing the progression of apoptosis by immunoblotting of PARP and PARP cleavage products in control and FLASH knockdown cells following the procedure outlined in panel B. (**D**) Immunofluorescence assay of caspase-3 activation (*Materials and Methods*) in HT1080 cells transfected with FLASH or control siRNA for 48 hours with additional 6 hours treatment with 100 ng/ml FasL. (**E**) Light micrographs of HT1080 cells transfected with FLASH and control siRNA for 72 hours and then stimulated with FasL for 16 hours.

RNA interference of FLASH expression did not induce apoptosis in HT1080 cells in the absence of apoptotic signals at all times tested up to 72 hours. However, apoptosis in the FLASH depleted cells proceeded much more rapidly when the Fas receptor was activated as compared to the cells transfected with control siRNA. A time course over six hours following stimulation of the Fas receptor (Figure 4A) clearly showed that both caspase 8 and the executioner caspase, caspase 3, are activated by proteolytic cleavage much more rapidly than the control cells. Similar results were obtained for caspase 8 when the receptor was activated by the recombinant human Fas ligand (Figure 4B). Quantification of the bands on the gel (Figure 4B) indicated that after three hours, there was an approximately eleven fold increase in the activation of caspase 8 in cells depleted of FLASH. After six hours, there was still an approximately three fold higher caspase-8 activity when FLASH was knocked down. There appeared to be a slight depletion of coilin six hours post stimulation of the receptor when FLASH expression was suppressed, an observation that may suggest that a fraction of the FLASH may be associated with coilin containing Cajal bodies. The significantly more rapid degradation of the anti-apoptotic protein, poly ADP-ribose polymerase (PARP), a hallmark of the early stages of apoptosis, in cells lacking FLASH was a further indication that FLASH depleted cells are more sensitive to Fas mediated apoptosis (Figure 4C). Immunofluorescence micrographs of cells stained with antibodies directed against cleaved caspase-3 (Figure 4D) were taken 6 hours following stimulation of HT1080 cells with Fas antibodies. The micrographs showed that caspase-3 activation had progressed more rapidly in cells depleted of FLASH. After 16 hours, most of the cells lacking FLASH had either died or were in advanced stages of apoptosis (Figure 4E). Similar results were obtained for MCF-10A cells (data not shown).

### The Intracellular level of FLASH decreases during apoptosis

**Figure 5:**
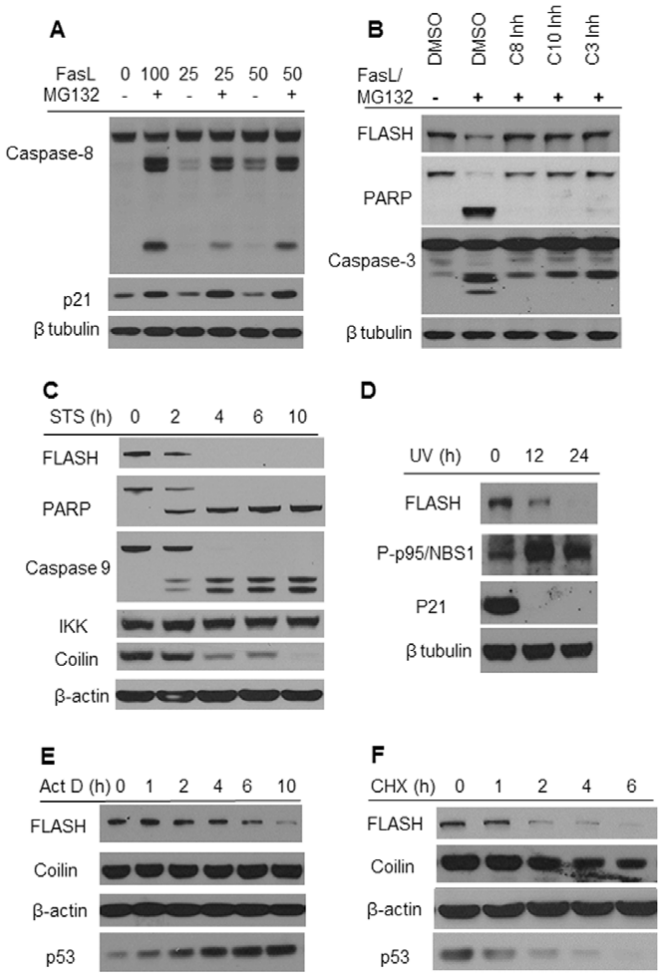
The Intracellular level of FLASH decreases during apoptosis. (**A**) The proteasome inhibitor MG132 potentiates caspase 8 activation induced by FasL in HT1080 cells. HT1080 cells were treated with the indicated concentration of FasL with or without 10 µM MG132 for 4 hours. The activation of caspase-8 was monitored by immunoblotting of the total cell lysates using caspase 8 antibodies. β-tubulin served as a loading control and p21, a protein with a short half-life, was a control showing that MG132 effectively blocks proteasomal activity. (**B**) FLASH was down-regulated following induction of apoptosis. HT1080 cells were either pretreated with the vehicle (DMSO) or caspase 3, 8 and 10 inhibitors for 30 minutes and then induced into apoptosis by exposure to 100 ng/ml FasL and 10 µM MG132 for 4 hours. The relative intracellular levels of FLASH, PARP, intact and cleaved, and caspase-3 were determined by immunoblotting. β-tubulin served as a loading control. (**C**) FLASH was also downregulated following induction of apoptosis in HeLa cells by exposure to 1 µM staurosporine for the indicated times. The cell lysates were analyzed by immunoblotting of FLASH, caspase-9, IKK, coilin and β-actin. (**D**) Apoptosis was induced by exposure to UV light (*Materials and Methods*). The cells were harvested 12 hours and 24 hours following a 5 minute UV exposure. The relative levels of FLASH, phospho-p95/NBS1, an indicator of DNA damage, P21 and β-tubulin were determined by immunoblotting. (**E**) Protein synthesis was blocked by incubating HT1080 cells with 50 µg/ml cycloheximide (CHX) for the indicated times and the relative level of FLASH, coilin, β-actin and p53 was determined by immunoblotting. (**F**) The relative levels of the same proteins as in panel (E) were determined by immunoblotting following inhibition of RNA transcription by exposure of HT1080 cells to 1 µg/ml actinomycin D for the indicated times.

Anti-apoptotic proteins are usually rapidly degraded once apoptosis has been initiated. The stability of FLASH was monitored during apoptosis under conditions where proteasomal degradation was blocked. MG132 is a potent proteasome inhibitor and significantly augments the rate at which many cell types progress through apoptosis perhaps as a consequence of blocking the proteasomal degradation of pro-apoptotic proteins [24]. The accelerated cleavage of caspase-8 indicated that MG132 also promotes apoptosis of HT1080 cells (Figure 5A). Moreover, the intracellular level of P21, a protein which turns over very rapidly, significantly increased confirming that proteasomal degradation had been inhibited.

The intracellular level of FLASH, like the anti-apoptotic protein, PARP, rapidly decreased during apoptosis in cells stimulated by FasL and MG132 (Figure 5B). The extensive cleavage of pro-caspase-3 served as a marker of apoptosis. However, the degradation of FLASH was completely arrested by the potent caspases 3, 8 and 10 inhibitors (Figure 5B), indicating that FLASH, like PARP, was degraded by caspases once apoptosis was underway. Similar results were obtained when apoptosis was initiated with staurosporine (Figure 5C). The time course following exposure to staurosporine showed that FLASH and PARP, as well as the Cajal body component, coilin, rapidly disappeared from the cell extract as apoptosis progressed. In this experiment, IKK and β-actin served as negative controls. FLASH was also degraded during apoptosis resulting from exposure to UV light (Figure 5D). DNA damage was confirmed by immunoblotting of phospho-p95/NBS1, a protein that is part of a complex that is phosphorylated by ATM in response to DNA breaks [25]. Thus, FLASH is rapidly degraded during the early stages of apoptosis independent of the induction method.

FLASH mRNA (Figure 5E) was found to be relatively stable with a half-life of approximately 6 hours as indicated by exposing the cells to the transcriptional inhibitor, actinomycin D. In contrast, exposure of the cells to cycloheximide, an inhibitor of protein synthesis (Figure 5F) suggests that the FLASH protein turns over rapidly (half-life 2–3 hours) raising the possibility that down-regulation of FLASH levels could be due to proteasomal degradation. However, since in the presence of MG132, FLASH degradation was blocked by the specific caspase inhibitors (Figure 5B), it is more likely that FLASH is degraded by caspases during apoptosis.

### Anti-apoptotic proteins were down regulated when FLASH was depleted

**Figure 6:**
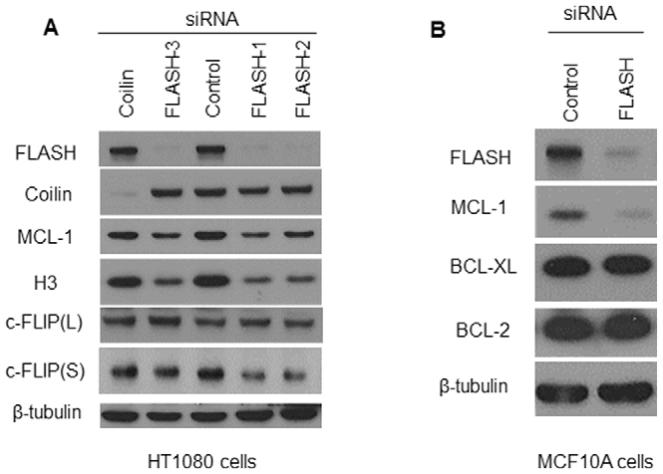
Effect of FLASH knockdown on the level of anti-apoptotic proteins. (**A**) HT1080 cells were transfected with 3 different FLASH siRNAs for 72 hours. Coilin and the scrambled siRNA served as controls. The intracellular level of FLASH, coilin, MCL-1, histone H3 and the long and short isoforms of cFLIP, cFLIP (L) and cFLIP (S), respectively, were determined by immunoblotting using the corresponding antibodies. β-tubulin served as a loading control. (**B**) Following the same protocol, MCF-10A cells were transfected with siRNA directed against FLASH or with control siRNA. Cell extracts were prepared 72 hours following transfection and the cell lyates were subjected to immunoblotting using antibodies directed against the indicated proteins.

The effect of silencing FLASH gene expression on several proteins implicated in apoptosis was assessed in HT1080 and MCF-10A cells. Both coilin siRNA and scrambled siRNA served as controls and three different FLASH siRNAs were tested. None of the proteins tested were affected by coilin siRNA or scrambled siRNA. Histone H3 levels were clearly reduced in FLASH depleted cells consistent with the result showing that FLASH knockdown causes cell cycle arrest (Figure 6A) by down regulating the synthesis of histones [8]. FLASH knockdown significantly reduced the intracellular levels of two anti-apoptotic proteins, MCL1 and the short isoform of c-FLIP, but not c-FLIP long, in HT1080 (Figure 6A). MCL-1 levels were also decreased by FLASH knockdown in MCF-10A cells (Figure 6B), but the level of BCL-XL was unaffected.

### Suppression of apoptosis by FLASH is p53 dependent

**Figure 7:**
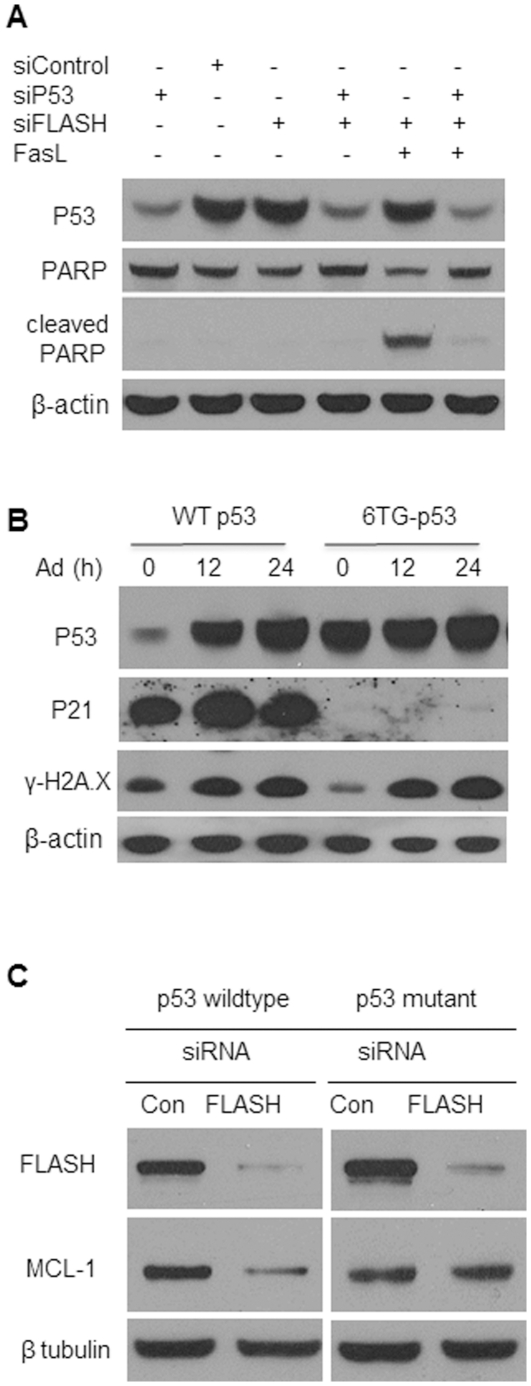
The effect of FLASH knockdown on apoptosis was dependent on p53. (**A**) HT1080 cells were transfected with scrambled siRNA (siControl), FLASH siRNA (siFLASH) and p53 siRNA (siP53) or co-transfected with both FLASH siRNA and p53 siRNA for 48 hours. Apoptosis was then induced by incubation with 100 ng/ml FasL for an additional 4 hours. Immunoblotting using p53 antibodies showed that p53 was effectively knocked down with siP53 in the presence and absence of siFLASH. Upon stimulation with the FasL, the increase in apoptosis in cells lacking FLASH was abolished in cells depleted of both FLASH and p53. (**B**) The effect of DNA damage incurred by exposure to adriamycin on the relative intracellular level of p53 and p21. Two isogenic cell lines, HT1080 (wildtype p53) and HT1080-6TG (p53 mutant), were treated with 200 ng/ml adriamycin for the indicated times. The intracellular level of p53 and p21 was determined by immunoblotting. The level of p-Histone H2A.X (Ser139) was used to monitor the progressive DNA damage induced by adriamycin treatment. β-actin served as a loading control. (**C**) The wild type HT1080 and HT1080-6TG cells (p53 mutant) were transfected, as in panel B, with siRNA against FLASH and the scrambled siRNA (Con) for 72 hours. The intracellular levels of FLASH and MCL-1 were determined by immunoblotting. β-tubulin served as a loading control.

RNA interference was used to reduce the level of p53 in HT1080 cells by approximately 80%. As expected, p53 knockdown (Figure 7A, lane 1) did not induce significant apoptosis judging from the observation that PARP was not degraded. Similarly, depleting the cells of FLASH or both FLASH and p53 (lanes 3 and 4) did not result in apoptosis. However, FLASH knock down cells with wild type 53 rapidly progressed through apoptosis following stimulation with the Fas ligand (lane 5). In contrast, stimulation of cells in which both FLASH and p53 were knocked down did not undergo apoptosis (lane 6), suggesting that a functional p53 is essential.

To confirm the involvement of p53 in promoting apoptosis upon FLASH knockdown, the expression of the anti-apoptotic protein MCL-1 was assessed in mutant HT1080 cells, 6TG-p53, which over express inactive p53. As shown previously [26], exposure of the cells to adriamycin, which produces double stranded breaks in DNA, results in up-regulation of p53 and as a result the up-regulation of its target, P21 (Figure 7B). These results confirm that although 6TG-p53 cells overproduce p53, the protein lacks transcriptional activity.

Stimulation of the Fas receptor in FLASH depleted wild type cells with a functional p53 gene resulted in down-regulation of MCL-1 (Figure 6 and 7B). However, in the 6TG-p53 cells, transfection with FLASH siRNA did not alter the intracellular level of MCL-1. Collectively, these results indicate that the suppression of apoptosis by FLASH is dependent on transcriptionally active, p53.

## Discussion

In accord with previous studies [10], we find that FLASH is a nuclear protein localized within nuclear bodies, primarily but not exclusively within histone gene clusters, where it co localizes with NPAT. A fraction of FLASH was associated with other nuclear bodies although colocalization with PLM bodies appeared to be minimal. We also found that depletion of FLASH lead to a decrease in histone H3 in the cell and arrest in the S phase of the cell cycle.

An unanticipated result of this study was that FLASH was found to be anti-apoptotic, whereas previous work indicated that it promotes apoptosis. Imai et al [1] observed that over expression of FLASH resulted in an approximately 25% increase in apoptosis as judged by the altered morphology of the cells. It may be significant that our studies were conducted with the Type II cells in which activation of the mitochondrial pathway is paramount [3].

Similarly, Milovic-Holm et al. [7] found that FLASH was pro-apoptotic although the mechanism proposed was quite different than that suggested by Imai et al. [1]. They found that depletion of FLASH by siRNA interference followed by induction of the Fas receptor with anti-Fas antibody for 7 hours resulted in an approximately 57% decrease in apoptosis. They proposed that activation of the receptor resulted in translocation of FLASH from nuclear bodies to the mitochondria where it activates caspase-8. These authors also conducted their studies with HT1080 cells, the same cells we used in this study. However, there may be differences in strain, P53 status, growth conditions, and methods of induction or antibody titer that could account for the differences in the results. In assessing the effect of FLASH knockdown on apoptosis, these authors [7] induced with the anti-Fas antibody but with far lower concentrations than we employed in our studies (0.025 µg/ml versus 1 µg/ml). Although differences in antibody titer cannot be ruled out, it is perhaps significant that these authors found that the suppression of apoptosis resulting from FLASH depletion was significantly impaired at higher concentrations of the Fas antibody.

The evidence presented here that FLASH, can also suppress apoptosis is compelling. The acceleration of FasL induced apoptosis by FLASH depletion was observed with three different siRNAs that targeted different regions of the FLASH mRNA. Transfection with FLASH siRNA did not induce apoptosis unless the Fas receptor was stimulated but growth was arrested in S phase. However, when FLASH was depleted, activation of the Fas receptor resulted in 1) more rapid activation of caspase 8 and caspase 3, 2) rapid degradation of PARP, 3) nuclear disintegration and DNA fragmentation and 4) the characteristic morphological changes of the cell. Moreover, like most anti-apoptotic proteins, FLASH was rapidly degraded once apoptosis has been irreversibly initiated. The current study is not the only report indicating that FLASH suppresses apoptosis. A siRNA screen identified 37 proteins essential for cell division [27]. FLASH is one of six proteins that when knocked down results in rapid cell death upon entry into mitosis.

There is precedence for apoptotic proteins playing a dual role. For example, DAXX, a nuclear protein that, like FLASH, is involved in both apoptosis and repression of gene expression has been variously reported to be both pro- and anti-apoptotic. Over expression of Daxx promotes Fas induced apoptosis by direct interaction with the Fas receptor [28] or via a nuclear pathway [29] suggesting that it is pro-apoptotic. The opposite conclusion was drawn from studies of Daxx-knockout embryos and embryonic stem cell lines [30] and by siRNA suppression of Daxx expression [31], [32]. These latter studies showed that depletion of Daxx resulted in an increased sensitivity to Fas mediated or stress induced apoptosis, suggesting an anti-apoptotic function. Whether Daxx promotes or suppresses apoptosis may be dependent on its modification by other signaling pathways. It was recently shown [33] that phosphorylation of Daxx by CK2 kinase promotes the binding of SUMO-1 and stress-induced apoptosis by down-regulation of anti-apoptotic regulatory proteins.

The involvement of FLASH in the activation of caspase-8 at the DISC [1] and at the mitochondria [7] is pro-apoptotic, but there are other functions ascribed to FLASH that would be expected to protect against entry into apoptosis.

Stimulation of TNF-α receptor elicits two opposing effects, apoptosis and activation of the anti-apoptotic transcription factor, NF-kappa B [34]. Suppression of FLASH expression has been shown to abolish TNF-α induced activation of NF-kappa B via a TRAF2 dependent pathway in HEK293 cells [21], an effect that would be expected to stimulate apoptosis. While the relationship between FLASH and Fas signaling has not been investigated, it has been shown that the stimulation of the Fas receptor also activates NF-kappa B in human bladder carcinoma T24 and Jurkat cells [35] and in SK-Hep1 hepatocellular carcinoma cells [36], so it is likely that FLASH has a comparable role in the Fas receptor signaling that would lead to suppression of apoptosis (Figure 1).

Another FLASH function consistent with an anti-apoptotic role is that it serves as an important coactivator of cMyb [16], [17], a transcription factor generally considered to promote growth and survival (Figure 1). c-Myb is a major target of glycogen synthase kinase 3β (GSK3β). Inhibition of GSK3β results in ubiquitin mediated degradation of cMyb and the induction of apoptosis by inhibiting the expression of BCL2 and survivin [37]. Survivin, a caspase inhibitor [38], may not be relevant since it is expressed primarily during G2 and FLASH knockdown arrest cell cycle progression in S phase [39]. BCL-2 is a potent inhibitor of apoptosis that blocks the function of the death inducing protein Bak at the mitochondrial membrane. However, we did not find a significant decrease in the BCL-2 levels when FLASH was depleted suggesting that it also does not play a role in the suppression of apoptosis by FLASH.

In agreement with Barcaroli et al [8], we found that FLASH colocalizes with NPAT in histone gene clusters. Moreover, the intracellular level of histone H3 is dramatically reduced upon FLASH depletion and the cells accumulate in S phase. Normally, cell cycle arrest in S-phase is not sufficient to induce apoptosis. However, the failure to assemble functional nucleosomes to protect the newly synthesized DNA may accelerate the onset of apoptosis when the cells are stimulated by FasL.

Suppression of apoptosis by FLASH was found to be dependent on the transcription factor, p53. In cells depleted of endogenous p53 by siRNA interference, knock down of FLASH had an appreciably smaller stimulatory effect on apoptosis when the Fas receptor was activated (Figure 5B). Similarly, in HT1080 cells harboring a mutant p53, FLASH knockdown did not significantly enhance the apoptotic response. P53 is known to regulate the expression of several pro- and anti-apoptotic proteins [40]. Previous studies suggested that P53 down-regulates the expression of the anti-apoptotic protein, MCL-1 up to 30-fold [40]. In this study, depletion of FLASH resulted in significant reduction in the level of MCL-1 in the cell. The lower concentration of MCL-1 would be expected to relieve its inhibitory effect on the formation of the mitochondrial channel and promote apoptosis.

The other anti-apoptotic protein that we found down regulated by FLASH depletion is the short isoform of c-FLIP (cellular FLICE inhibitory protein) which acts directly at the Fas death-inducing DISC complex inhibiting caspase-8 activation and Fas receptor mediated apoptosis [41]. In hepatocellular carcinoma cells, c-FLIP is the major regulator of cell death and survival. In addition to inhibiting caspase activation, c-FLIP is a potent inhibitor of apoptosis by inhibiting the activation of NF-kappa B [42]. The function of the long isoform c-FLIP has been controversial, but recent studies indicate that it can be either pro- or anti-apoptotic depending on the circumstances [41], [43].

The precise role that FLASH may play regulation of intracellular level of MCL-1 and c-FLIP short remains to be determined. Previous studies showed that FLASH promotes both activation and repression of gene expression depending on the specific gene under consideration [8], [15], [16], [17], [18], [19], [20], [21] and thus it may modulate the level of these anti-apoptotic proteins. Alternatively, both MCL-1 and c-FLIP short are short lived proteins [44], [45], so the reduced intracellular levels of these proteins upon FLASH depletion may result from the destabilization of these proteins as a consequence of cell cycle arrest in S phase.

While there are several functional connections between FLASH and various key factors in the apoptotic pathways, the mechanism by which FLASH suppresses apoptosis remains to be deciphered. Of particular interest, is the identification of the switch that determines whether FLASH functions to promote or suppress apoptosis. Very little is known regarding the regulation of FLASH function, however, FLASH has been shown to interact with the SUMO-conjugation enzyme, Ubc9 [46]. Sumoylation attenuates the transcriptional activity of FLASH as measured by the Gal4 tethering assay. Given the size and complexity of FLASH, other regulatory mechanisms are likely to be discovered.
